# Supplementary material for: Prevalence of non-communicable diseases risk factors and their determinants: Results from STEPS survey 2019, Nepal
Source: PLoS One. 2021 Jul 30;16(7):e0253605. doi: 10.1371/journal.pone.0253605 (PMC8323895; doi:10.1371/journal.pone.0253605)
Supplement: S1 File — (PDF) [file pone.0253605.s001.pdf]

ANNEX 2 : QUESTIONNAIRE

Noncommunicable Disease Risk Factors STEPS Survey, Nepal 2019

Survey instrument

(Core and Expanded)

The WHO STEP wise approach to noncommunicable disease risk factor surveillance (STEPS) 2019

Survey Information

Location and Date	Response	Code
Interviewer ID<br>*Must be between 1 to 30.*	└─┴─┴─┘	I3
PSU ID<br>*PSU code must be between 101 to 137 or 201 to 237 or 301 to 337 or 401 to 437 or 501 to 537 or 601 to 637 or 701 to 737.*	└─┴─┴─┴─┴─┴─┘	I1
Date of completion of the instrument<br>*Fill automatically.*	└─┴─┘ └─┴─┘ └─┴─┴─┴─┘<br>dd mm year	I4
Time of interview<br>(24-hour clock)<br>*Fill automatically.*	└─┴─┘ : └─┴─┘<br>hrs mins	I7
Family Surname<br>*It will fill automatically, please check before editing*		I8
First Name<br>*It will fill automatically, please check before editing*		I9
Contact number of respondents<br>*Must be in 10 digits; Put zero before number if it is less then 10 digits.*	└─┴─┘ └─┴─┘ └─┴─┘ └─┴─┘ └─┴─┘<br>Enter 88, if refused and 99, if not available	I10
Consent has been read and obtained	Yes 1<br>No 2 **If NO, END**	I5

Step 1 Demographic Information

Question	Response	Code
Sex (*Record Male / Female as observed)*<br>*It will fill automatically, please check before editing*	Male 1<br>Female 2	C1
What is your date of birth?<br>*Don't Know 77 77 7777*	└─┴─┘ └─┴─┘ └─┴─┴─┴─┘ *If Known, Goto C4*<br>dd mm year	C2
How old are you?	Years └─┴─┘	C3
In total, how many years have you spent at school and in full-time study (excluding pre-school) [COUNT FROM GRADE 1]?<br>*Should be between 0 - 25 years*	Years └─┴─┘ if 0 then go to C6	C4
What is the **highest level of education** you have completed?	No formal schooling 1<br>Less than primary school 2<br>Primary school completed 3<br>Secondary school completed 4<br>High school completed (+2, intermediate, PCL) 5<br>Bachelor level completed 6<br>Post graduate degree 7<br>Refused 88	C5
What is your *ethnic* **background**?<br>*[REFER CASTE CLASSIFICATION CARD – CC1]*	Dalit 1<br>Disadvantaged Janajati 2<br>Disadvantaged Non-Dalit Tarai caste group 3<br>Religious Minorities 4<br>Relatively advantaged janajati 5<br>Upper caste Group 6<br>Others 7<br>Refused 88	C6
What is your **marital status**?	Never married 1<br>Currently married 2<br>Separated 3<br>Divorced 4<br>Widowed 5<br>Cohabitating 6<br>Refused 88	C7
Which of the following best describes your **main work** status over the past 12 months?	Government employee 1<br>Non-government employee 2<br>Self-employed 3<br>Non-paid 4<br>Student 5<br>Homemaker 6<br>Retired 7<br>Unemployed (able to work) 8 go to C9x1<br>Unemployed (unable to work) 9 go to C9x1<br>Others 10<br>Refused 88	C8/ C8Other
Are you currently working as Health Care Worker such as doctor, dental surgeon, public health administrator/ officers, nurse, pharmacist, health assistants, physiotherapists, auxiliary health workers, ANM, Midwife, FCHV?	Yes 1<br>No 2	C8x1
Are you currently working as a teacher/ instructor/ faculty/ lecturer/ professor in any school/ college/ university/ academic institutes?	Yes 1<br>No 2	C8x2

In total, how many persons live in this household (including infants)?	└─┴─┘	C9x1
Is any lady in the house currently pregnant?	Yes 1<br>No 2<br>Don't know 77<br>Refuse 88	C10x
**Please ask/ observe - whether this household or any person who lives in the household has the following items:**		
a. Electricity	Yes 1 | No 2	C11xa
b. Radio	Yes 1 | No 2	C11xb
c. Television	Yes 1 | No 2	C11xc
d. Landline	Yes 1 | No 2	C11xd
e. Mobile phone	Yes 1 | No 2	C11xe
f. Computer	Yes 1 | No 2	C11xf
g. Refrigerator	Yes 1 | No 2	C11xg
h. Inverter	Yes 1 | No 2	C11xh
i. Bed	Yes 1 | No 2	C11xi
j. Sofa	Yes 1 | No 2	C11xj
k. Table	Yes 1 | No 2	C11xk
l. Fan	Yes 1 | No 2	C11xl
m. Chair	Yes 1 | No 2	C11xm
n. Watch / Clock	Yes 1 | No 2	C11xn
o. Bicycle	Yes 1 | No 2	C11xo
p. Motor cycle / Scooter	Yes 1 | No 2	C11xp
q. Car / Truck / Jeep / Tractor	Yes 1 | No 2	C11xq
r. Dhiki /Jato	Yes 1 | No 2	C11xr
s. Animal drawn cart	Yes 1 | No 2	C11xs
t. Domestic animal like Cow / Buffalo / Goat	Yes 1 | No 2	C11xt
What is the main material of the roof of the main house? [RECORD OBSERVATIONS]		
**Natural roofing**		
No roof	1	C12x/ C12xOther
Thatched/Palm leaf	2	
**Rudimentary Roofing**		
Rustic mat	3	
Bamboo	4	
Wood Planks	5	
Cardboard	6	
**Finished roofing**		
Metal/Galvanized sheet	7	
Wood	8	
Calamine/cement fiber	9	
Ceramic tiles	10	
Cement	11	
Roofing singles	12	
Other (Specify)	13	

Step 1 Behavioural Measurements

Tobacco Use

Now I am going to ask you some questions about tobacco use.

Question	Response	Code
Do you **currently** smoke any **tobacco** products, such as cigarettes, bidis, cigars, pipes, hukahs, or tamakhus? *(USE SHOWCARDS 1a)*	Yes 1<br>No 2 *If No, go to T8*	T1
Do you currently smoke tobacco products **daily**?	Yes 1<br>No 2	T2
How old were you when you **first started** smoking?	Age (years)<br>Don't know 77 └─┴─┘ *If Known, go to T5a/T5aw*	T3
Do you remember how long ago it was?<br>*(RECORD ONLY 1, NOT ALL 3)*<br>*Don't know 77*	In Years └─┴─┘ *If Known, go to T5a/T5aw*	T4a
	OR in Months └─┴─┘ *If Known, go to T5a/T5aw*	T4b
	OR in Weeks	T4c
On average, **how many** of the following products do you smoke **each day/week?**<br>*(FOR CIGARETTES, INTERVIEWER NEED TO VERIFY THIS IS THE NUMBER OF CIGARETTES' NOT PACKS)*<br>*(RECORD EITHER DAILY OR WEEKLY, BUT NOT BOTH, IF LESS THAN DAILY, RECORD WEEKLY)*<br>*(RECORD FOR EACH TYPE)*<br>*(USE SHOWCARDS 1a)*<br>*Don't Know 7777*	DAILY↓ WEEKLY↓	
	Manufactured cigarettes └─┴─┴─┴─┘ └─┴─┴─┴─┘	T5a/T5aw
	Hand-rolled cigarettes └─┴─┴─┴─┘ └─┴─┴─┴─┘	T5b/T5bw
	Pipes full of tobacco └─┴─┴─┴─┘ └─┴─┴─┴─┘	T5c/T5cw
	Cigars, cheroots, cigarillos └─┴─┴─┴─┘ └─┴─┴─┴─┘	T5d/T5dw
	Bidi └─┴─┴─┴─┘ └─┴─┴─┴─┘	T5e/T5ew
	Hukka sessions └─┴─┴─┴─┘ └─┴─┴─┴─┘	T5f/T5fw
	Other └─┴─┴─┴─┘ └─┴─┴─┴─┘<br>*If Other, go to T5other, else go to T6*	T5g/T5gw
	Other (please specify): └─┴─┴─┴─┴─┴─┘	T5other/ T5otherw
During the past 12 months, have you tried to **stop smoking**?	Yes 1<br>No 2	T6
During any visit to a doctor or other health worker in the past 12 months, were you advised to quit smoking tobacco?	Yes 1 *If T2=Yes, go to T12; if T2=No, go to T9*<br>No 2 *If T2=Yes, go to T12; if T2=No, go to T9*<br>No visit during the past 12 months 3 *If T2=Yes, go to T12; if T2=No, go to T9*	T7
In the past, did you **ever smoke** any tobacco products? *(USE SHOWCARDS 1a)*	Yes 1<br>No 2 *If No, go to T12*	T8
In the past, did you **ever** smoke **daily**?	Yes 1 *If T1=Yes, go to T12, else go to T10*<br>No 2 *If T1=Yes, go to T12, else go to T10*	T9
How old were you when you **stopped** smoking?	Age (years)<br>Don't Know 77 └─┴─┘ *If Known, go to T12*	T10
How **long ago** did you stop smoking?<br>*(RECORD ONLY 1, NOT ALL 3)*<br>*Don't Know 77*	Years ago └─┴─┘ *If Known, go to T12*	T11a
	OR Months ago └─┴─┘ *If Known, go to T12*	T11b
	OR Weeks ago └─┴─┘ *If Known, go to T12*	T11c

Do you **currently use** any **smokeless tobacco** products such as *snuff, chewing tobacco, nasal snuffs, Khaini, surti, gutkha*?<br>*(USE SHOWCARDS 1b)*	Yes 1<br>No 2 *If No, go to T15*	T12
Do you **currently use smokeless tobacco** products such as snuff, chewing tobacco, nasal snuffs, khaini, surti, gutkha **daily?**	Yes 1<br>No 2 *If No, go to T14aw*	T13
On average, how many **times a day/week** do you use<br>*(RECORD EITHER DAILY OR WEEKLY, BUT NOT BOTH, IF LESS THAN DAILY, RECORD WEEKLY)*<br>*(RECORD FOR EACH TYPE)*<br>*(USE SHOWCARDS 1b)*<br>*Don't Know 7777*	DAILY↓ WEEKLY↓	
	Snuff, by mouth └┴┴┴┘ └┴┴┴┘	T14a/ T14aw
	Snuff, by nose └┴┴┴┘ └┴┴┴┘	T14b/ T14bw
	Chewing tobacco └┴┴┴┘ └┴┴┴┘	T14c/ T14cw
	Betel leaves with tobacco (Jarda pan) └┴┴┴┘ └┴┴┴┘	T14d/ T14dw
	Betel, quid without tobacco (Sada pan) └┴┴┴┘ └┴┴┴┘	T14e/ T14ew
	Gutkha └┴┴┴┘ └┴┴┴┘	T14f/ T14fw
	Surti └┴┴┴┘ └┴┴┴┘	T14g/ T14gw
	Khaini └┴┴┴┘ └┴┴┴┘	T14h/ T14hw
	Other └┴┴┴┘ └┴┴┴┘<br>*If Other, go to T14other, if T13=No, go to T16, else go to T17*	T14i/ T14iw
	Other (please specify): └┴┴┴┴┴┘<br>*If T13=No, go to T16, else go to T17*	T14other/ T14otherw
In the **past**, did you **ever use** smokeless tobacco products such as *snuff, chewing tobacco, nasal snuff, khaini, surti, gutka*?	Yes 1<br>No 2 *If No, go to T17*	T15
In the **past**, did you **ever use** smokeless tobacco products such as *snuff, chewing tobacco, nasal snuff, khaini, surti, gutka* **daily**?	Yes 1<br>No 2	T16
During the past 12 months, have you tried to **stop using smokeless tobacco products?**	Yes 1<br>No 2	Tx1
During any visit to a doctor or other health worker in the past 12 months, were you advised to **quit smokeless tobacco?**	Yes 1<br>No 2<br>No visit during the past 12 months 3	Tx2
During the past 12 months, what did you do to try and **stop smoking or smokeless tobacco?** [Multiple answer]<br>If T6=yes or Tx1=yes	1. Counseling by any health care workers<br>2. Nicotine replacement therapy, such as the patch or gum<br>3. Traditional medicine like ayurvedic, homeopathy, unani, naturopathy etc.<br>4. A quit line or telephone support line<br>5. Try to quit without assistance<br>6. Other (Specify)	Tx3
During the past 30 days, did someone smoke **in your home in your presence?**	Yes 1 if yes, then go to T17x<br>No 2	T17
How often does anyone smoke in your home? Would you say daily, weekly, monthly, or less than monthly?	Daily 1<br>Weekly 2<br>Monthly 3<br>Less than monthly 4<br>Don't know 5	T17x
During the past 30 days, did someone smoke in closed areas **where you work** (in the building, in a work area or a specific office)?	Yes 1<br>No 2<br>Don't work in a closed area 3	T18

In the past 30 days, did anyone smoke inside following places when you visited those places? Restaurants / Bars / Canteens / Hotel	Yes 1<br>No 2<br>Didn't visit 77	Tx5a
Public transport such as bus/taxi/tempo including bus stands and ticketing counter	Yes 1<br>No 2<br>Didn't use public transport 77	Tx5b
School/College/University/hostels	Yes 1<br>No 2<br>Didn't visit 77	Tx5c
Health care facilities (Hospitals/Health Post/Primary Health Care Centers/ clinics)	Yes 1<br>No 2<br>Didn't visit 77	Tx5d

Electronic Cigarettes

The next questions are about using electronic cigarettes. Electronic cigarettes include any product that uses batteries or other methods to produce a vapor which contains nicotine. They have various other names such as e-cigarette, vape-pen, e-shisha, e-pipes.

Question	Response		Code
Before today, have you ever heard of electronic cigarettes?	Yes<br>No<br>Refused	1<br>2 [If 'No' go to TP1a]<br>88 [go to TP1a]	EC1
Which one of the following is an electronic cigarette? *[USE SHOWCARDS 1c]*	Pipes full of tobacco<br>E-cigarette<br>Shisha<br>Hukka	1<br>2<br>3<br>4	EC2
Do you currently use electronic cigarettes?	Yes, Daily<br>Less than daily<br>Not at all<br>Refused	1 [go to TP1a]<br>2 [go to TP1a]<br>3<br>88	EC3
Have you ever, even once, used an electronic cigarette?	Yes<br>No<br>Refused	1<br>2<br>88	EC4

Tobacco Policy

You have been asked questions on tobacco consumption before. The next questions ask about tobacco control policies. They include questions on your exposure to the media and advertisement, on cigarette promotions, health warnings and cigarette purchase.

Question	Response	Code
During the past 30 days, have you **noticed information about the dangers of smoking** cigarettes, bidis or other tobacco products that encourages quitting through the following media? *(RECORD FOR EACH)* Newspapers or magazines	Yes 1<br>No 2<br>Don't know 77	TP1a
Television	Yes 1<br>No 2<br>Don't know 77	TP1b
Radio	Yes 1<br>No 2<br>Don't know 77	TP1c
Internet/Websites	Yes 1<br>No 2<br>Don't use internet 77	TP1d

In the last 30 days, have you seen any advertisements or signs promoting the cigarettes/bidis or any other smokeless tobacco products such as chewing tobacco / gutkha / surti / khaini on following medias? *(RECORD FOR EACH* Newspapers or magazines	Yes 1<br>No 2<br>Don't know 77	TPx1
Television	Yes 1<br>No 2<br>Don't know 77	TPx2
Radio	Yes 1<br>No 2<br>Don't know 77	TPx3
Internet / Websites	Yes 1<br>No 2<br>Don't know 77	TPx4
Billboards/posters/wall painting	Yes 1<br>No 2<br>Don't know 77	TPx5
During the past 30 days, have you noticed any **advertisements** or **signs** promoting cigarettes/bidis or any other tobacco products in stores where cigarettes are sold?	Yes 1<br>No 2<br>Don't know 77	TP2
During the past 30 days, have you noticed any of the following types of cigarette promotions? *(RECORD FOR EACH)* Free samples of cigarettes	Yes 1<br>No 2<br>Don't know 77	TP3a
Cigarettes at sale prices	Yes 1<br>No 2<br>Don't know 77	TP3b
Coupons for cigarettes	Yes 1<br>No 2<br>Don't know 77	TP3c
Free gifts or special discount offers on other products when buying cigarettes	Yes 1<br>No 2<br>Don't know 77	TP3d
Clothing or other items with a cigarette brand name or logo	Yes 1<br>No 2<br>Don't know 77	TP3e
Cigarette promotions in the mail	Yes 1<br>No 2<br>Don't know 77	TP3f
During the past 30 days, did you notice any **health warnings on cigarette/bidis/smokeless tobacco product packages**?	Yes 1<br>No 2 *go to TP6*<br>Did not see any tobacco packages 3 *go to TP6*<br>Don't know 77 *go to TP6*	TP4
The next questions TP5 – TP7 are to be asked for current smokers or current users of smokeless tobacco products		
During the past 30 days, have warning labels on cigarette/bidis/smokeless tobacco product packages led you to **think about quitting**?	Yes 1<br>No 2<br>Don't know 77	TP5
The last time you bought manufactured cigarettes for yourself, **how many cigarettes** did you buy in total?	Number of cigarettes └┴┴┴┴┘<br>Don't know or Don't smoke or purchase manuf. Cigarettes enter 7777 *If selected, end section*	TP6
In total, **how much money** did you pay for this purchase?	Amount └┴┴┴┴┘<br>Don't know 7777<br>Refused 8888	TP7
Last time you bought cigarette for yourself, did you buy loose cigarettes, packets or something else how did you buy it?	Loose Cigarettes 1<br>Packet 2<br>Others specify	TPx6/ TPx6others

Alcohol Consumption

The next questions ask about the consumption of alcohol.

Question	Response		Code
Have you **ever** consumed an alcoholic drink such as beer, wine, spirits fermented cider or *jaad, chyang, raksi, aila or tungba*? *(USE SHOWCARDS 2a)*	Yes 1<br>No 2	*If No, go to A16*	A1
Have you consumed an alcoholic drink within the **past 12 months**?	Yes 1<br>No 2	If Yes, go to A4	A2
What are the reasons you stopped alcohol during past 12 months? (MULTIPLE RESPONSE)	Health reason<br>Family Pressure<br>Can't afford/No money to buy<br>Just wanted to stop<br>Spiritual/religious reasons<br>Advice of your doctor or other health worker<br>Other (Specify)	1 *go to AP1*<br>2 *go to AP1*<br>3 *go to AP1*<br>4 *go to AP1*<br>5 *go to AP1*<br>6 go to *AP1*<br>7 *go to AP1*	Ax1/ Ax1others
During the past 12 months, **how frequently** have you had at least one standard alcoholic drink? *(READ RESPONSES)* *(USE SHOWCARDS 2b)*	Daily<br>5-6 days per week<br>3-4 days per week<br>1-2 days per week<br>1-3 days per month<br>Less than once a month	1<br>2<br>3<br>4<br>5<br>6	A4
Have you consumed any alcohol within the **past 30 days**?	Yes 1<br>No 2	*If No, go to A13*	A5
What is the type of alcohol do you usually or most often consume? *(SELECT ONLY ONE)*	Beer<br>Wine<br>Spirit (Whiskey / Vodka / Gin)<br>Jaad<br>Rakshi<br>Aila<br>Other	1<br>2<br>3<br>4<br>5<br>6<br>8	Ax2/ Ax2Other
During the past 30 days, on how many **occasions** did you have at least one standard alcoholic drink? *(USE SHOWCARDS 2b)*	Number<br>Don't know 77	└─┴─┘ *if A6=0 goto A8*	A6
During the past 30 days, when you drank alcohol, how many **standard drinks on average** did you have during one drinking occasion? *(USE SHOWCARDS 2b)*	Number<br>Don't know 77	└─┴─┘	A7
During the past 30 days, what was the **largest number** of standard drinks you had on a single occasion, counting all types of alcoholic drinks together?	Largest number<br>Don't Know 77	└─┴─┘	A8
During the past 30 days, how many times did you have six or more Standard drinks in a single drinking occasion?	Number of times<br>Don't Know 77	└─┴─┘	A9
During each of the **past 7 days**, how many standard drinks did you have each day? *(USE SHOWCARDS 2b)* *Don't Know 77*	Monday	└─┴─┘	A10a
	Tuesday	└─┴─┘	A10b
	Wednesday	└─┴─┘	A10c
	Thursday	└─┴─┘	A10d
	Friday	└─┴─┘	A10e

	Saturday └─┴─┘	A10f
	Sunday └─┴─┘	*A10g*
I have just asked you about your consumption of alcohol during the past 7 days. The questions were about alcohol in general, while the next questions refer to your consumption of homebrewed alcohol, alcohol brought over the border/from another country, any alcohol not intended for drinking or other untaxed alcohol. Please only think about these types of alcohol when answering the next questions.		
During the **past 7 days**, did you consume any **homebrewed** alcohol like chyang, rakshi, jaad, aila, tungba, any alcohol **brought over the border/from another country**, any alcohol **not intended for drinking** or other **untaxed** alcohol? *(USE SHOWCARDS 2c)*	Yes 1<br>No 2 *If No, go to A13*	A11
On average, **how many standard drinks** of the following did you consume **during the past 7 days**? *(USE SHOWCARDS 2c)* *Don't Know 77*	Homebrewed spirits like aila, raksi └─┴─┘	A12a
	Homebrewed beer or wine, like jaad, chyang, tungbaa └─┴─┘	A12b
	Alcohol brought over the border/from another country └─┴─┘	A12c
	Alcohol not intended for drinking, like alcohol-based medicines, like cough syrup, perfumes, after shaves └─┴─┘	A12d
	Others untaxed alcohol in the country Specify	A12e
**Alcohol Consumption if, A2=1**		
During the **past 12 months,** how often have you found that you were not able to stop drinking once you had started?	Daily or almost daily 1<br>Weekly 2<br>Monthly 3<br>Less than monthly 4<br>Never 5	A13
During the **past 12 months,** how often have you failed to do what was normally expected from you because of drinking?	Daily or almost daily 1<br>Weekly 2<br>Monthly 3<br>Less than monthly 4<br>Never 5	A14
During the **past 12 months,** how often have you needed a first drink in the morning to get yourself going after a heavy drinking session?	Daily or almost daily 1<br>Weekly 2<br>Monthly 3<br>Less than monthly 4<br>Never 5	A15
During the **past 12 months**, have you had family problems or problems with your partner due to **someone else's** drinking?	Yes, more than monthly 1<br>Yes, monthly 2<br>Yes, several times but less than monthly 3<br>Yes, once or twice 4<br>No 5	A16

Alcohol Policy and programs

You have been asked questions on alcohol consumption before. The next questions ask about alcohol control policies and programs. They include questions on your exposure to the media and advertisement, on alcohol promotions, enforcement of bans or comprehensive restrictions on alcohol advertising, drunk driving countermeasures, restricting physical availability, health warnings and alcohol purchases.

How easy or difficult it is for you to **obtain alcohol** for drinking? (if A1=yes)	Very easy<br>Easy<br>Difficult<br>Very difficult<br>Don't know/don't drink alcohol	1<br>2<br>3<br>4<br>77	AP1
Has it become less or more **affordable** to obtain alcohol now compared to two years before? (if A1=yes)	More affordable than before<br>Same as before<br>Less affordable than before<br>Don't know/don't drink alcohol	1<br>2<br>3<br>77	AP2
During last 30 days, have you **driven a vehicle** after intake or	Yes	1	AP3

under the influence of alcohol?	No 2<br>I don't drive 3	
(if A1=yes)<br>During last 12 months, have you been **stopped/ checked by traffic police** for alcohol while driving?	Yes 1<br>No 2<br>I don't drive 77<br>Refused 88	AP4
During the last 30 days, have you noticed any **advertisements or signs promoting** *beer, wine, any other spirits etc. on television, newspapers/magazine, radio, Billboards, Point of sale or, local magazines, local cinema/films?*	Yes 1<br>No 2<br>Don't know 77	AP5
When you go to sports events, fairs, concerts, community events, or social gatherings, how often do you see **advertisements**, free beer/alcohol or discounted sale of alcohol?	Not attended any such gathering 1<br>Never 2<br>Rarely 3<br>Sometimes 4<br>Most of the time 5<br>Always 6	AP6
During the past 30 days, did you see or hear any messages on television, radio, billboards, posters, newspapers, magazines, or movies, internet, social media that **discourages you to drink alcohol** or informs you about health dangers of drinking alcohol?	Yes 1<br>No 2	AP7
During the past 30 days, did anyone **refuse to sell** you *beer, arrack, wine & other spirits etc.* because of your age?	Yes 1<br>No 2<br>I did not try to buy 3	AP8

Diet

The next questions ask about the fruits and vegetables that you usually eat. I have a nutrition card here that shows you some examples of local fruits and vegetables. Each picture represents the size of a serving. As you answer these questions please think of a typical week in the last year.

In a typical week, on how many days do you **eat fruit**?<br>*(USE SHOWCARDS 3a)*	Number of days<br>Don't Know 77 └─┴─┘ *If Zero days, go to D3*	D1
How many **servings** of fruit do you eat on **one** of those days?<br>(*USE SHOWCARDS 3b)*	Number of servings<br>Don't Know 77 └─┴─┘	D2
In a typical week, on how many days do you **eat vegetables**?<br>*(USE SHOWCARDS 3c)*	Number of days<br>Don't Know 77 └─┴─┘ *If Zero days, go to Dx1*	D3
How many **servings** of vegetables do you eat on one of those days? *(USE SHOWCARDS 3d)*	Number of servings<br>Don't know 77 └─┴─┘	D4
What do you think is the desirable or recommended number of fruit and vegetable servings one should eat every day to be healthy?	Number of servings<br>Don't know 77 └─┴─┘	Dx1

Dietary salt

The next questions ask about your knowledge, attitudes and behaviour towards dietary salt. Dietary salt includes ordinary table salt, unrefined salt such as sea salt, iodised salt and salty sauces such as soya sauce or fish sauce. The following questions are on adding salt to food right before you eat it, how food is prepared in your home, eating processed foods that are high in salt such as instant noodles (chau chau), salted potato chips, salty biscuits, canned fish, dry meat, titaura, preserved pickle, bhujia, papad etc. and on controlling your salt intake. Please answer the questions even if you consider yourself to eat a diet low in salt.

How often do you **add salt** to your food right before you eat it or as you are eating it (adding extra salt from the table)?<br>*(SELECT ONLY ONE)*<br>*(USE SHOWCARDS 4a)*	Always 1<br>Often 2<br>Sometimes 3<br>Rarely 4<br>Never 5<br>Don't know 77	D5a
How often do you **add salt sauce such as soya sauce or other sauces** to your food right before you eat it or as you are eating?<br>*(SELECT ONLY ONE)*<br>*(USE SHOWCARDS 4b)*	Always 1<br>Often 2<br>Sometimes 3<br>Rarely 4<br>Never 5<br>Don't know 77	D5b
How often do you eat **processed food high in salt**?	Always 1	D7

Processed food high in salt means foods that have been altered from their natural state, such as packaged salty snacks (such as chau chau, salty buscuits, lays, kur kure, nimkeen, chips, titura, bhujiya), pappad canned salty food including *aachar* and preservatives, salty food prepared at a fast food restaurant, cheese, processed meat, dried fish, salty fish etc. *(USE SHOWCARDS 4c)*	Often 2<br>Sometimes 3<br>Rarely 4<br>Never 5<br>Don't know 77	
**How much salt** do you think you consume?	Far too much 1<br>Too much 2<br>Just the right amount 3<br>Too little 4<br>Far too little 5<br>Don't know 77	D8a
**How much salty sauce such as soya sause** do you think you consume?	Far too much 1<br>Too much 2<br>Just the right amount 3<br>Too little 4<br>Far too little 5<br>Don't know 77	D8b
How important is it to you to **lower salt** in your diet?	Very important 1<br>Somewhat important 2<br>Not at all important 3<br>Don't know 77	D9
What is the maximum amount of salt do you think a person should take in a day from all sources? [In Teaspoonful (TSF)]	└─┴─┘ Teaspoonful<br>Don't know 77	Dx2
What do you think that too much salt in your diet can do to your health? [Multiple response]	Nothing, more salt is good for health 1<br>Increase blood pressure 2<br>Kidney disease 3<br>Asthma 4<br>Cancer 5<br>Tuberculosis 6<br>Other specify 7<br>Don't Know 77	Dx3/ Dx3other
Currently are you doing anything on regular basis to control salt intake?	Yes 1<br>No 2 *go to Dx5*<br>Don't know 77 *go to Dx5*	Dx4
Do you do any of the following on a regular basis to **control your salt intake**? *(RECORD FOR EACH)*		
Avoid /minimize consumption of processed foods such as achaar or papad	Yes 1<br>No 2	D11a
Look at the salt or sodium content on food labels	Yes 1<br>No 2	D11b
Buy low salt/sodium alternatives	Yes 1<br>No 2	D11c
Use spices other than salt when cooking	Yes 1<br>No 2	D11d
Avoid eating foods prepared outside of home.	Yes 1<br>No 2	D11e
Eat meals without adding extra salt at the table	Yes 1<br>No 2	D11f
Cook meals such as rice or bread without adding salt	Yes 1<br>No 2	D11g
Others	Yes 1<br>No 2	D11h

Other (please specify)	└┴┴┴┴┴┴┘		D11other
The next questions ask about the **oil or fat** that is most often used for meal preparation in your household, and about meals that you eat outside a home.			
What types of oil or fat is most often used for meals preparation in your household	Mustard oil<br>Refined vegetable oil<br>Lard or suet<br>Butter ghee<br>Noodles oil<br>Vanaspati ghee<br>Others (specify)<br>Nothing in particular<br>Not used<br>Don't know	1<br>2<br>3<br>4<br>5<br>6<br>7<br>8<br>9<br>77	Dx5/ Dx5other
On an average, how many meals (breakfast, lunch or dinner) **per week** do you eat that were not prepared at a home?	Number<br>Don't know	└┴┴┘<br>77	Dx6

Physical Activity

Next, I am going to ask you about the time you spend doing different types of physical activity in a typical week. Please answer these questions even if you do not consider yourself to be a physically active person. Think first about the time you spend doing work. Think of work as the things that you have to do such as paid or unpaid work, study/training, household chores, harvesting food/crops, fishing or hunting for food, seeking employment. *[Insert other examples if needed].* In answering the following questions 'vigorous-intensity activities' are activities that require hard physical effort and cause large increases in breathing or heart rate, 'moderate-intensity activities' are activities that require moderate physical effort and cause small increases in breathing or heart rate.

Work

Does your work involve **vigorous-intensity** activity that causes large increases in breathing or heart rate like *carrying or lifting heavy loads, digging, ploughing cycling rikshaw or construction work* for at least 10 minutes continuously? *(USE SHOWCARDS 5a)*	Yes<br>No	1<br>2 *If No, go to P 4*	P1
In a typical week, on how many days do you do vigorous-intensity activities as part of your work?	Number of days	└┘ Enter 77, if not known	P2
How much time do you spend doing vigorous-intensity activities at work on a typical day?	Hours: minutes	└┴┘ : └┴┘<br>hrs mins<br>Enter 77, if not known	P3 (a-b)
Does your work involve **moderate-intensity** activity that causes small increases in breathing or heart rate *such as brisk walking, carrying light loads, manual washing clothes, mopping of floor, gardening at home* for at least 10 minutes continuously? *(USE SHOWCARDS 5b)*	Yes<br>No	1<br>2 *If No, go to P 7*	P4
In a typical week, on how many days do you do moderate-intensity activities as part of your work?	Number of days	└┘ Enter 77, if not known	P5
How much time do you spend doing moderate-intensity activities at work on a typical day?	Hours: minutes	└┴┘ : └┴┘<br>hrs mins<br>Enter 77, if not known	P6 (a-b)

Travel to and from places

The next questions exclude the physical activities at work that you have already mentioned. Now I would like to ask you about the usual way you travel to and from places. For example, to work, for shopping, to market, to place of worship.

Do you walk or use a bicycle *(pedal cycle)* for at least 10 minutes continuously to get to and from places?	Yes<br>No	1<br>2 *If No, go to P 10*	P7
In a typical week, on how many days do you walk or bicycle for at least 10 minutes continuously to get to and from places?	Number of days	└┘ Enter 77, if not known	P8
How much time do you spend walking or bicycling for travel on a typical day?	Hours: minutes	└┴┘ : └┴┘<br>hrs mins<br>Enter 77, if not known	P9 (a-b)

Recreational activities		
The next questions exclude the work and transport activities that you have already mentioned. Now I would like to ask you about sports, fitness and recreational activities (leisure).		
Do you do any **vigorous-intensity** sports, fitness or recreational (leisure) activities that cause large increases in breathing or heart rate *[running or Football]* for at least 10 minutes continuously? *(USE SHOWCARDS 5c)*	Yes 1<br>No 2 *If No, go to P 13*	P10
In a typical week, on how many days do you do vigorous-intensity sports, fitness or recreational *(leisure)* activities?	Number of days └─┘ Enter 77, if not known	P11
How much time do you spend doing vigorous-intensity sports, fitness or recreational activities on a typical day?	Hours: minutes └─┴─┘ : └─┴─┘ hrs mins<br>Enter 77, if not known	P12 (a-b)
Do you do any **moderate-intensity** sports, fitness or **recreational (leisure) activities** that cause a small increase in breathing or heart rate *[brisk walking, cycling, swimming, volleyball, badminton, Yoga]* for at least 10 minutes continuously? *(USE SHOWCARDS 5d)*	Yes 1<br>No 2 *If No, go to P16*	P13
In a typical week, on how many days do you do moderate-intensity sports, fitness or recreational *(leisure)* activities?	Number of days └─┘ Enter 77, if not known	P14
How much time do you spend doing moderate-intensity sports, fitness or recreational *(leisure)* activities on a typical day?	Hours: minutes └─┴─┘ : └─┴─┘ hrs mins<br>Enter 77, if not known	P15 (a-b)
**Sedentary behaviour**		
The following question is about sitting or reclining at work, at home, getting to and from places, or with friends including time spent sitting at a desk, sitting with friends, travelling in car or bus, reading, playing cards or watching television, but does not include time spent sleeping *(USE SHOWCARDS 5e)*		
How much time do you usually spend sitting or reclining on a typical day?	Hours: minutes └─┴─┘ : └─┴─┘ hrs mins<br>Enter 77, if not known	P16 (a-b)

History of Raised Blood Pressure

Have you **ever** had your **blood pressure measured** by a doctor or other health worker?	Yes 1<br>No 2 *If No, go to H6*	H1
Have you **ever** been told by a doctor or other health worker that you **have raised blood pressure** or hypertension?	Yes 1<br>No 2 *If No, go to H6*	H2a
Were you first **told in the past 12 months**?	Yes 1<br>No 2	H2b
Have you **ever been told** to **take a medicine** by a doctor or health workers **for raised blood pressure**?<br>[Appear only if H2a=yes]	Yes 1<br>No 2	Hx1
Have you **ever taken drugs/medications** for raised blood pressure prescribed by a doctor/health worker?<br>[Appear only if H2a=yes]	Yes 1<br>No 2 *[If No, go to Hx2]*	Hx1a
In the **past two weeks**, have you **taken any drugs** (medication) for raised blood pressure prescribed by a doctor or other health worker?<br>[Appear only if H2a= yes and Hx1a=yes]	Yes 1<br>No 2	H3
Which type of drugs are you taking for treatment of raised blood pressure? [Multiple response]<br>(Use BP drug list card)<br>(Observe the drugs for those who respond for H3=yes)	Angiotensin converting enzyme inhibitors (ACEIs) 1<br>Calcium channel blockers (CCBs) 2<br>Angiotensin-receptor blockers 3<br>Beta-blockers 4<br>Diuretics 5<br>Others (specify generic name) 6	Hx1b
Where do you usually go for treatment or advice for your raised blood pressure?<br>[Multiple Response]	Govt. Tertiary level hospital 1<br>Govt. Regional and sub-regional hospital 2<br>Govt. District hospital 3<br>Govt. Primary Health Care centre 4<br>Govt. Health Post 5	Hx2<br>Hx2other

[Appear only *If H2a=yes]*	NGO run/Community hospital 6<br>Private hospital 7<br>Private Clinic 8<br>Ayurvedic, homeopathic or naturopathic hospital/clinic 9<br>Medical shops/Pharmacies 10<br>Other (specify) 11<br>Don't know 77	
Where do you usually get your drugs for raised blood pressure?<br>[Multiple Response]<br>[Appear only *If Hx1a=yes or H3=yes]*	Govt. Tertiary level hospital 1<br>Govt. Regional and sub-regional hospital 2<br>Govt. District hospital 3<br>Govt. Primary Health Care centre 4<br>Govt. Health Post 5<br>NGO run/Community hospital 6<br>Private hospital 7<br>Private Clinic 8<br>Ayurvedic, homeopathic or naturopathic hospital/clinic 9<br>Medical shops/Pharmacies 10<br>Other (specify) 11<br>Don't know 77	Hx3/ Hx3Other
What is the most important reason for which you are not currently taking medications for raised blood pressure or hypertension?<br>[Appear only if H2a=yes and (Hx1a=no or H3=no)]	Don't think drug is necessary 1<br>Got side effects 2<br>Afraid of side effects 3<br>Too expensive 4<br>Blood pressure got normal 5<br>Medicine not available 6<br>Medicine not advised by doctor 7<br>Other (specify) 8	Hx4/ Hx4other
Have you ever seen a traditional healer like Dhami / Jhakri/ Purohit / Lama / Gubaju / Matas for raised blood pressure or hypertension?	Yes 1<br>No 2 go to H6	H4
Are you currently taking any herbal or traditional remedy for your raised blood pressure?	Yes 1<br>No 2	H5
**History of Diabetes**		
Have you **ever had your blood sugar measured** by a doctor or other health worker?	Yes 1<br>No 2 *If No, go to H12*	H6
Have you **ever** been **told** by a doctor or other health worker that you have **raised blood sugar** or diabetes?	Yes 1<br>No 2 *If No, go to H12*	H7a
Were you first **told in the past 12 months**?	Yes 1<br>No 2	H7b
Have you ever been **told to take a medicine** by a doctor or health workers for raised blood sugar or diabetes?<br>[Appear only if H7a=yes]	Yes 1<br>No 2	Hx5
Have you **ever taken drugs**/medications for diabetes prescribed by a doctor/health worker?<br>[Appear only if H7a=yes]	Yes 1<br>No 2 *([If No, go to Hx6]*	Hx5a
In the **past two weeks**, have you **taken any drugs** (medication) for diabetes prescribed by a doctor or other health worker? [Appear only if H7a=yes and Hx5a=yes]	Yes 1<br>No 2 go to Hx6	H8
Are you currently taking insulin for diabetes prescribed by a doctor or other health worker?<br>[Appear only if H7a=yes]	Yes 1<br>No 2	H9
Where do you usually go for treatment or advice for diabetes?<br>[Multiple Response]	Govt. Tertiary level hospital 1<br>Govt. Regional and sub-regional hospital 2<br>Govt. District hospital 3<br>Govt. Primary Health Care centre 4	*Hx6/ Hx6other*

[Appear only *If H7a=yes]*	Govt. Health Post	5	
	NGO run/Community hospital	6	
	Private hospital	7	
	Private Clinic	8	
	Ayurvedic, homeopathic or naturopathic hospital/clinic	9	
	Medical shops/Pharmacies	10	
	Others (specify)	11	
	Don't know	77	
Where do you usually get your drugs for diabetes? [Multiple Response] [Appear only *If Hx5a = yes or H8 = yes or H9 = yes]*	Govt. Tertiary level hospital	1	*Hx7/ Hx7other*
	Govt. Regional and sub-regional hospital	2	
	Govt. District hospital	3	
	Govt. Primary Health Care centre	4	
	Govt. Health Post	5	
	NGO run/Community hospital	6	
	Private hospital	7	
	Private Clinic	8	
	Ayurvedic, homeopathic or naturopathic hospital/clinic	9	
	Medical shops/Pharmacies	10	
	Others (specify)	11	
	Don't know	77	
What is the most important reason for which you are not currently taking medications for raised blood sugar or diabetes? [Appear only if, H7a = yes and (Hx5a=no or H8)]	Don't think drug is necessary	1	*Hx8/ Hx8other*
	Got side effects	2	
	Afraid of side effects	3	
	Too expensive	4	
	Diabetes got normal	5	
	Medicine not available	6	
	Medicine not advised	7	
	Other (specify)	8	
Have you ever seen a traditional healer like Dhami/ Jhakri/ Purohit/ Lama/ Qubaju/ Matas for diabetes or raised blood sugar?	Yes	1	H10
	No	2 go to H12	
Are you currently taking any herbal or traditional remedy for your diabetes?	Yes	1	H11
	No	2	
**History of Raised Total Cholesterol**			
Have you ever had your **cholesterol** (fat levels in your blood) **measured by a doctor** or other health worker?	Yes	1	H12
	No	2 *If No, go to H17*	
Have you ever been **told by a doctor** or other health worker that **you have raised cholesterol**?	Yes	1	H13a
	No	2 *If No, go to H17*	
Were you first **told in the past 12 months**?	Yes	1	H13b
	No	2	
Have you ever been **told to take a medicine** by a doctor or health workers for **raised cholesterol**?	Yes	1	Hx9
	No	2	
Have you **ever taken drugs**/medications for raised blood cholesterol prescribed by a doctor/health worker?	Yes	1	Hx10
	No	2 *If No, go to Hx11*	
In the **past two weeks**, have you **taken any oral treatment** (medication) for raised total cholesterol prescribed by a doctor or other health worker?	Yes	1	H14
	No	2	
Where do you usually **go for treatment** or advice for your raised total cholesterol? [Multiple Response]	Govt. Tertiary level hospital	1	Hx11/ Hx11other
	Govt. Regional and sub-regional hospital	2	
	Govt. District hospital	3	

[Appear only *If H13a=yes]*	Govt. Primary Health Care centre 4<br>Govt. Health Post 5<br>NGO run/Community hospital 6<br>Private hospital 7<br>Private Clinic 8<br>Ayurvedic, homeopathic or naturopathic hospital/clinic 9<br>Medical shops/Pharmacies 10<br>Others (Specify) 11<br>Don't know 77	
Where do you usually **get your drugs** for raised total cholesterol?<br><br>[Multiple Response]<br><br>[Appear only *If Hx10 = yes or H14 = yes]*	Govt. Tertiary level hospital 1<br>Govt. Regional and sub-regional hospital 2<br>Govt. District hospital 3<br>Govt. Primary Health Care centre 4<br>Govt. Health Post 5<br>NGO run/Community hospital 6<br>Private hospital 7<br>Private Clinic 8<br>Ayurvedic, homeopathic or naturopathic hospital/clinic 9<br>Medical shops/Pharmacies 10<br>Others (Specify) 11<br>Don't know 77	Hx12/ Hx12other
What is the most important reason for which you are not currently taking medications for raised blood cholesterol?<br><br>Appear only if,<br>[H13a = yes and H14 = no]	Don't think drug is necessary 1<br>Got side effects 2<br>Afraid of side effects 3<br>Too expensive 4<br>Cholesterol got normal 5<br>Medicine not available 6<br>Medicine not advised 7<br>Other (specify) 8	Hx13/ Hx13other
Have you ever seen a traditional healer like Dhami/ Jhakri/ Purohit/ Lama/ Qubaju/ Matas for raised cholesterol?	Yes 1<br>No 2 go to H17	H15
Are you currently taking any herbal or traditional remedy for your raised cholesterol?	Yes 1<br>No 2	H16
**History of Cardiovascular Diseases**		
Have you ever had a **heart attack or chest pain** from heart disease (angina) or a stroke (cerebrovascular accident or incident)?	Yes 1<br>No 2	H17
Are you **currently taking aspirin** regularly to prevent or treat heart disease?	Yes 1<br>No 2	H18
Are you currently taking **statins** (Lovastatin/Simvastatin/Atorvastatin or any other statin) regularly to prevent or treat heart disease?	Yes 1<br>No 2	H19
**Lifestyle Advice**		
During the past 12 months, have you visited a doctor or other health worker?	Yes 1<br>No 2 *If No and C1=1, go to O2*<br>*If No and C1=2, go to CX1*	H20

During any of your visits to a doctor or other health worker in the past 12 months, were you advised to do any of the following? *(RECORD FOR EACH)*		
Quit using tobacco or don't start	Yes 1<br>No 2	H20a
Reduce salt in your diet	Yes 1<br>No 2	H20b
Eat at least five servings of fruit and/or vegetables each day	Yes 1<br>No 2	H20c
Reduce fat in your diet	Yes 1<br>No 2	H20d
Start or do more physical activity	Yes 1<br>No 2	H20e
Maintain a healthy body weight or lose weight	Yes 1<br>No 2	H20f
Reduce sugary beverages in your diet	Yes 1 *If C1=1 go to O2 and C1=2 go to Cx1*<br>No 2 *If C1=1 go to O2 and C1=2 go to Cx1*	H20g

Cervical Cancer Screening (for women only)

The next question asks about cervical cancer prevention. Screening tests for cervical cancer prevention can be done in different ways, including Visual Inspection with Acetic Acid/vinegar (VIA), pap smear and Human Papillomavirus (HPV) test. VIA is an inspection of the surface of the uterine cervix after acetic acid (or vinegar) has been applied to it. For both pap smear and HPV test, a doctor or nurse uses a swab to wipe from inside your vagina, take a sample and send it to a laboratory. It is even possible that you were given the swab yourself and asked to swab the inside of your vagina. The laboratory checks for abnormal cell changes if a pap smear is done, and for the HP virus if an HPV test is done.

Have you ever had a test for cervical cancer, using any of these methods described above?	Yes 1 go to CX2<br>No 2<br>Don't know 77	CX1
At what age were you **first tested** for cervical cancer?	Age └─┴─┘<br>Don't know 77<br>Refused 88	CX2
When was your **last (most recent) test** for cervical cancer?	Less than 1 year ago 1<br>1-2 years ago 2<br>3-5 years ago 3<br>More than 5 years ago 4<br>Don't know 77<br>Refused 88	CX3
What is the **main reason** you had your **last** test for cervical	Part of a routine exam 1<br>Following up on abnormal or inconclusive result 2<br>Recommended by healthcare provider 3<br>Recommended by other source 4<br>Experiencing pain or other symptoms 5<br>Other (Specify) 6<br>Don't know 77<br>Refused 88	CX4/ CX4other
**Where** did you receive your last test for cervical cancer?	Govt. Tertiary level hospital 1<br>Govt. Regional and sub-regional 2<br>Govt. District hospital 3<br>Govt. Primary Health Care centre 4<br>Govt. Health Post 5	CX5/ CX5other

Question	Response	Code	Skip	Variable
	NGO run/Community hospital	6		
	Private hospital	7		
	Private Clinic	8		
	Other (specify)	9		
	Don't know	77		
What was the result of your **last (most recent)** test for cervical	Did not receive result	1	*If CX6=1, go to O2*	CX6
	Normal / Negative	2	*If CX6=2, go to O2*	
	Abnormal /Positive	3		
	Suspect cancer	4		
	Inconclusive	5		
	Don't know	77		
	Refused	88		
Did you have any follow-up visits because of your test results?	Yes	1		CX7
	No	2		
	Don't know	3		
	Refused	4		
Did you receive any treatment to your cervix because of your test results?	Yes	1		CX8
	No	2		
	Don't know	3		
**Oral Health**				
The next questions I will ask about your oral health status and related behaviours.				
How would you describe the **state of your teeth?**	Excellent	1		O2
	Very Good	2		
	Good	3		
	Average	4		
	Poor	5		
	Very Poor	6		
	Don't Know	77		
How would you describe the **state of your gums?**	Excellent	1		O3
	Very Good	2		
	Good	3		
	Average	4		
	Poor	5		
	Very Poor	6		
	Don't know	77		
Do you have any **removable dentures**?	Yes	1		O4
	No	2	*If No, go to O6*	
Which of the following removable dentures do you have? *(RECORD FOR EACH)*				
An upper jaw denture	Yes	1		O5a
	No	2		
A lower jaw denture	Yes	1		O5b
	No	2		
During the past 12 months, did your teeth, gums or mouth cause any **pain, swelling, bleeding or discomfort**?	Yes	1		O6
	No	2		
How long has it been since you last **saw a dentist**?	Less than 6 months	1		O7
	6-12 months	2		
	More than 1 year but less than 2	3		
	2 or more years but less than 5 years	4		
	5 or more years	5		
	Never received dental care	6	*If Never, go to O9*	
What was the **main reason for your last visit** to the dentist?	Consultation / advice	1		O8/ O8other
	Pain or trouble with teeth, gums or	2		
	Treatment / Follow-up treatment	3		
	Routine check-up treatment	4		
	Other (Specify)	5	*If Other, go to O9other*	
How **often do you clean** your teeth?	Never	1	*If Never, go to O13a*	O9
	Once a month	2		
	2-3 times a month	3		
	Once a week	4		
	2-6 times a week	5		

	Once a day<br>Twice or more a day	6<br>7	
Do you use **toothpaste** to clean your teeth?	Yes<br>No	1<br>2 *If No, go to O12a*	O10
Do you use **toothpaste** containing **fluoride**?	Yes<br>No<br>Don't know	1<br>2<br>77	O11
Do you use any of the following to **clean your teeth on usual basis**?<br>*(RECORD FOR EACH)*			
Toothbrush	Yes<br>No	1<br>2	O12a
Wooden toothpicks (Neem stick)	Yes<br>No	1<br>2	O12b
Plastic toothpicks	Yes<br>No	1<br>2	O12c
Thread (Dental floss)	Yes<br>No	1<br>2	O12d
Charcoal	Yes<br>No	1<br>2	O12e
Chewstick / Miswak/ Dattiwan	Yes<br>No	1<br>2	O12f
Other	Yes<br>No	1 *If Yes, go to O12other*<br>2	O12g
Other (please specify)	└─┴─┴─┴─┴─┴─┴─┴─┘		O12other
Have you **experienced any of the following problems** during the past 12 months because of the **state of your teeth, gums or mouth**?<br>*(RECORD FOR EACH)*			
Difficulty in chewing foods	Yes<br>No	1<br>2	O13a
Difficulty with speech/trouble pronouncing words	Yes<br>No	1<br>2	O13b
Bleeding from gums	Yes<br>No	1<br>2 If no, go to O13e	O13c
When does your gums normally bleed?	On brushing<br>On eating hard food<br>Spontaneously	1<br>2<br>3	*O13d*
Swelling from gums	Yes<br>No	1<br>2	*O13e*
Embarrassed about appearance of teeth	Yes<br>No	1<br>2	O13f
Have a red and white patch in mouth	Yes<br>No	1<br>2	O13g
Have a persistent wound and /or swelling in mouth for more than 3 weeks	Yes<br>No	1<br>2	O13h
Days not at work because of teeth or mouth	Yes<br>No	1<br>2	O13i
Difficulty doing usual activities	Yes<br>No	1<br>2	O13j
Having difficulty in opening mouth	Yes<br>No	1<br>2	O13k
Are you currently suffering from dental caries?	Yes<br>No<br>Don't know	1<br>2<br>3	*Ox1*
Did you visit health facilities (hospital/PHCC/HP) because of dental caries?<br>*(Should appear if yes to any questions O13a to O13k)*	Yes<br>No	1<br>2 If no, go to *Ox4*	*Ox2*
Where do you usually go for oral health problems?<br>*(If, Ox2=yes)*	Govt. Tertiary level hospital<br>Govt. Regional and sub-regional<br>Govt. District hospital<br>Govt. Primary Health Care Centre<br>Govt. Health Post<br>NGO run/Community hospital<br>Dental homes/hospital	1<br>2<br>3<br>4<br>5<br>6<br>7	*Ox3/ Ox3other*

	Private hospital	8		
	Private Clinic	9		
	Ayurveda, homeopathic or	10		
	Medical shops/Pharmacies	11		
	Other (Specify)	....		
	Don't know	77		
Why you did NOT take treatment or advice? *(If, Ox1=yes and Ox2=no)*	Not serious enough to required treatment	1		*Ox4/ Ox4other*
	Did not know how/where to get treatment	2		
	Too expensive	3		
	Didn't have time	4		
	Health Centre too far away	5		
	Poor service quality	6		
	Fear of procedure	7		
	Family member did not allow it	8		
	Other specify			
	Refused	88		
**Violence and Injury**				
**Injury**				
The next questions ask about different experiences and behaviors that are related to road traffic injuries.				
In the past 30 days, how often did you use a seat belt when you were the driver or passenger of a motor vehicle?	All of the time	1		V1
	Sometimes	2		
	Never	3		
	Have not been in a vehicle in past 30 days	4		
	No seat belt in the car I usually drive	5		
	Don't Know	77		
	Refused	88		
In the past 30 days, how often did you wear a helmet when you drove or rode as a passenger on a motorcycle or motor-scooter?	All of the time	1		V2
	Sometimes	2		
	Never	3		
	Have not been on a motorcycle or motor-scooter in past 30 days	4		
	Do not have a helmet	5		
	Don't Know	77		
	Refused	88		
In the past 12 months, have you been involved in a road traffic crash as a driver, passenger, pedestrian, or cyclist?	Yes (as driver)	1		V3
	Yes (as passenger)	2		
	Yes (as pedestrian)	3		
	Yes (as a cyclist)	4		
	No	5	*If No, go to V5*	
	Don't know	77	*If don't know, go to V5*	
	Refused	88	*If Refused, go to V5*	
Did you have any injuries in this road traffic crash which required medical attention?	Yes	1		V4
	No	2		
	Don't know	77		
	Refused	88		
The next questions ask about the most serious accidental injury you have had in the past 12 months.				
In the past 12 months, were you injured accidentally, other than the road traffic crashes which required medical attention?	Yes	1		V5
	No	2	*If No, go to V8*	
	Don't know	77	*If don't know, go to V8*	
	Refused	88	*If Refused, go to V8*	
Please indicate which of the following the cause of this injury was.	Fall	1		V6
	Burn	2		
	Poisoning	3		
	Cut	4		
	Near-drowning	5		

	Animal bite	6	
	Other (specify)	7	
	Don't know	77	
	Refused	88	
	Other (please specify)	└─┴─┴─┴─┴─┴─┴─┘	V6other
Where were you when you had this injury?	Home	1	V7
	School	2	
	Workplace	3	
	Road/Street/Highway	4	
	Farm	5	
	Sports/athletic area	6	
	Other (specify)	7	
	Don't know	77	
	Refused	88	
	Other (please specify)	└─┴─┴─┴─┴─┴─┴─┘	V7other
**Unintentional Injury**			
The next questions ask about behaviours related to your safety and whether or not you drink alcohol while driving or being a passenger.			
In the past 30 days, how many times have you ridden in a motorized vehicle where the driver has had 2 or more alcoholic drinks?	Number of times	└─┴─┘	V10
	Don't Know	77	
	Refused	88	
**Violence**			
The following questions are about different experiences and behaviors that are related to violence.			
In the past 12 months, how many times were you in a violent incident in which you were injured and required medical attention?	Never	1 *If never, go to MHx1*	V11
	Rarely (1- 2 times)	2	
	Sometimes (3 – 5 times)	3	
	Often (6 or more times)	4	
	Don't know	77 *If don't know, go to MHx1*	
	Refused	88 *If Refused, go to MHx1*	
The next questions ask about the most serious violent incidence you have had in the past 12 months.			
Please indicate which of the following caused your most serious injury in the last 12 months.	Being shot with a firearm	1	V12
	A weapon (other than a firearm) was used by the person who injured me	2	
	Being injured without any weapon (slapped, pushed)	3	
	Don't know	77	
	Refused	88	
**Mental Health**			
Following questions relate to your stress level in different setting as per your subjective experience			
Do you have any of the following stress?			
Work/business Stress	No	1	MHx1
	Some	2	
	High	3	
General stress at home	No	1	MHx2
	Some	2	
	High	3	
Severe financial stress/Due to unemployment	Yes	1	MHx3
	No	2	
Stressful life events in past year which disturbed you a lot	Yes	1	MHx4
	No	2	

Joint and Back Pain		
In the past 12 months, did you ever experience followings (For question BK1 and BK2)		
Pain, aching, stiffness or swelling in or around the joint (like that arms, hands, legs or feet) which were not related to an injury and lasted for more than a month?	Yes 1<br>No 2	BK1
Stiffness in the joint (such as hands, legs) in the morning after getting up from bed, or after a long rest of the joint without movement?	Yes 1<br>No 2 (If No go to BK5)	BK2
How long does this stiffness last?<br>*READ CHOICES AND MARK AS APPROPRIATE*	About 30 minutes or less 1<br>More than 30 minutes 2	BK3
Does this stiffness go away after exercise or movement in the joint?	Yes 1<br>No 2	BK4
During the past 30 days, did you experience back pain (including disc problems) that prevented you from doing usual household chores or going for work?	Yes 1<br>No 2	BK5
During the past 30 days, did you experience severe headache that prevented you from doing usual household chores or going out for work?	Yes 1<br>No 2	BK6

Miscellaneous			
Are you member of any health insurance scheme?	Yes 1<br>No 2 go to Mx3		Mx1
What type of insurance scheme do you have?	*Swasthya Bimaa Karyakram* (provided by Government of Nepal) 1<br>Private Insurance 2<br>Community based health insurance 3<br>Others (Specify) 4		Mx2/ Mx2other
On an average how much do you usually **spend in a one month** for **care** (including travel to health facility, fees, medicines, medical test or any other related expenses) of your **chronic disease** (hypertension, diabetes, raised cholesterol etc.)? (for those who have been told hypertensive or diabetic or having raised cholesterol)	Rs.– └┘ └┘ └┘	Enter '77' if not known, or '88' if refused	Mx3

Step 2 Physical Measurements		
**Blood Pressure**		
Interviewer ID	└─┴─┴─┘	M1
Reading 1	Systolic (mmHg) └─┴─┴─┘	M4a
	Diastolic (mmHg) └─┴─┴─┘	M4b
	Beats per minute └─┴─┴─┘	M16a
Reading 2	Systolic (mmHg) └─┴─┴─┘	M5a
	Diastolic (mmHg) └─┴─┴─┘	M5b
	Beats per minute └─┴─┴─┘	M16b
Reading 3	Systolic (mmHg) └─┴─┴─┘	M6a
	Diastolic (mmHg) └─┴─┴─┘	M6b
	Beats per minute └─┴─┴─┘	M16c
During the past two weeks, have you been treated for raised blood pressure with drugs (medication) prescribed by a doctor or other health worker?	Yes 1<br>No 2	M7
**Height, Weight, Waist and Hip Circumference**		
**For women:** Are you pregnant?	Yes 1 *If Yes, go to End*<br>No 2	M8
Height	in Centimetres (cm) └─┴─┴─┘.└─┘	M11
Weight<br>*If too large for scale 666.6*	in Kilograms (kg) └─┴─┴─┘.└─┘	M12
Waist circumference	in Centimeters (cm) └─┴─┴─┘.└─┘	M14
Hip circumference	in Centimeters (cm) └─┴─┴─┘.└─┘	M15

Step 3 Biochemical Measurements		
**CORE: Blood Glucose**		
**Question**	**Response**	**Code**
Enter participant's ID (generated in Step 1 and QR code)	└─┴─┘	PID-3
During the past 12 hours have you had anything to eat or drink, other than water?	Yes 1<br>No 2	B1
Technician ID	└─┴─┴─┘	B2
Device ID	└─┴─┘	B3
Time of day blood specimen taken (24hour clock)	Hours: minutes └─┴─┘ : └─┴─┘ hrs mins	B4
Fasting blood glucose (if B1=no)	mg/dl └─┴─┴─┘ . └─┘	B5
Random blood glucose (if B1=yes)	mg/dl └─┴─┴─┘ . └─┘	B5x
Today, have you taken insulin or other drugs (medication) that have been prescribed by a doctor or other health worker for raised blood glucose?	Yes 1<br>No 2	B6
**CORE: Blood Lipids**		
Total cholesterol	mg/dl └─┴─┴─┘ . └─┘	B8
During the past two weeks, have you been treated for raised cholesterol with drugs (medication) prescribed by a doctor or other health worker?	Yes 1<br>No 2	B9
Had you been fasting prior to the urine collection?	Yes 1<br>No 2	B10
Time of day urine sample taken (24hour clock)	Hours: minutes └─┴─┘ : └─┴─┘ hrs mins	B13

Data will be key-in in the laboratory

Urinary sodium and creatinine		
Enter participant's ID (generated in Step 1) and QR code	└─┴─┘	PID-4
Lab ID	└─┴─┴─┘	B11
Urinary sodium	mmol/l	B14
Urinary creatinine	mmol/l	B15
