# Supplementary material for: Prevalence of non-communicable diseases risk factors and their determinants: Results from STEPS survey 2019, Nepal
Source: PLoS One. 2021 Jul 30;16(7):e0253605. doi: 10.1371/journal.pone.0253605 (PMC8323895; doi:10.1371/journal.pone.0253605)
Supplement: Attachment — Submitted filename: comments.docx [file pone.0253605.s003.docx]

**Introduction**

The rationale for reporting this study could be reframed better.

For ex. There is no link between the multi sectoral plan and current STEPS survey; linking these two might give better clarity to understand the need for present STEPS survey

**Methods**

Sampling methods adapted in previous STEPS surveys are different from the current survey. The change in context should get mentioned for the readers to compare and interpret these two surveys

As per methods details provided in the reference, the sample size was estimated to cover each province with equal number of participants (around 900 each). If it is so, why there is 20% participants from some province and 8% from some province. Please clarify.

As province and ecological belts were considered as independent variables across all NCD risk factors give a brief write up regarding the characteristics of these provinces and ecological belts which are relevant to understand the burden of NCD risk factors.

Though sample size and sampling methods were published elsewhere it is better to give a brief description on the sample size and selection of PSUs.

Under Ethical concern, for minors it require assent from the adolescents and consent from parents. Hence it needs to be clarified accordingly. Avoid the tern assent consent.

**Results:**

In this survey 15-29 years age group is predominant which is entirely different from the previous STEPS survey or Nepal population distribution based on age.

Please clarify how this over representation from this age group has happened.

Two decimals in results make the tables too crowded. It does not become reader friendly.

There is no display of gender disaggregated results within the province. But the discussion section presented in those lines.

Tobacco use and smokers are used inter changeably. Please use the term consistently

For fruits & vegetables intake, when everything is more than 97% sub group disaggregation does not help much (line no 221 to 223) could be avoided.

Table 2: As majority of the times the number in the sub group is going to remain same across all NCD risk factors it seems there is redundancy the way the n is represented. Instead of total number in the sub group it is preferred to give number with the particular risk factor present under the column n.

**Discussion**

Comparison of smoking with previous STEPS survey is incomplete.

The speculation for higher prevalence of smoking in Nepal is not justified well. (line No 268-274). Actually the prevalence in Nepal is more that should be explained by extent of lacunae in implementation of current tobacco legislative measures.

Adjusted prevalence ratio or adjusted relative risk has been calculated but it has been interpreted as if they were odds ratios. (line No 322-324, 334-335, 337,350, 353)

This study has the lot of scope to bring out the change or impact of previous strategic plan through comparison of previous STEPs survey with the current. In similar lines, if there is reduction or increase in the NCD risk factor level the reasons could have been explained better.
